# Glyphosate treatments for managing successional dynamics in beech bark disease-affected northern hardwood forests

**DOI:** 10.1371/journal.pone.0336126

**Published:** 2025-11-14

**Authors:** Mark Givelas, Adam Gorgolewski, Cameron Duckett, Adam R. Martin

**Affiliations:** 1 Department of Physical and Environmental Sciences, University of Toronto Scarborough, Scarborough, Ontario, Canada; 2 Haliburton Forest Research Institute, Haliburton Forest and Wild Life Reserve Ltd., Haliburton, Ontario, Canada; 3 Institute of Forestry and Conservation, John H Daniels Faculty of Architecture Landscape and Design, University of Toronto, Toronto, Ontario, Canada; University of the Punjab Quaid-i-Azam Campus: University of the Punjab, PAKISTAN

## Abstract

The spread of beech bark disease (BBD) in northern tolerant hardwood forests poses a significant forest management challenge. Extensive aboveground mortality in BBD-affected stands often leads to the rapid formation of high-density American beech (*Fagus grandifolia* Ehrh.) thickets, primarily driven by vegetative regeneration through root sprouting. These thickets can outcompete desirable species such as sugar maple (*Acer saccharum* L.), and negatively impact long-term forest structure and functions. This study evaluated the efficacy of post-harvest herbicide treatments—specifically the application of glyphosate to recently cut stumps and the use of “hack-and-squirt” application techniques on standing beech—to suppress vegetative beech regeneration. Over five years, beech regeneration was significantly lower in treatment plots, averaging 904 stems ha ⁻ ¹ (95% CI: 433−1,378 stems ha ⁻ ¹), compared to 1,741 stems ha-¹ (95% CI: 1,286–2,193 stems ha ⁻ ¹) in untreated control plots. Additionally, by five years post-harvest, glyphosate-treated plots supported higher densities of desirable tree species such as sugar maple, indicating that the intervention shifted species composition by reducing beech dominance. However this method had no significant effect on overall tree species richness or diversity, so while these treatments effectively suppress beech regeneration and promote successional trajectories in hardwood forests, they do not reduce tree diversity. By alleviating the competitive dominance of beech thickets, this management strategy is likely to mitigate the ecological and economic impacts associated with BBD, while maintaining or enhancing desirable tree species diversity.

## Introduction

Since its introduction from Europe to eastern Canada in the 1890s, beech bark disease (BBD) has devastated American beech (*Fagus grandifolia* Ehrh.) populations across North America, causing widespread and lasting impacts on forest ecosystems. This disease complex involves an invasive scale insect (*Cryptococcus fagisuga* Lind.) and a fungal pathogen (*Neonectria faginata* [Lohman et al.] Castl. and/or *N. ditissima* [Tul. & C. Tul.] Samuels & Rossman), where feeding by the scale insect predisposes the tree to fungal infection [1,2]. Together, these disease agents have led to extensive aboveground mortality of beech, with several direct and cascading ecological effects [3,4,5]. The infection rate among beech trees across North America is estimated to be between 80–95% [6], resulting in losses across environmental, economic, social, and cultural spheres. For example, more than two decades after the introduction and spread of BBD in Vermont, United States, an estimated 300 million board feet (707,921 m^3^) of economically viable beech were lost [7]. In addition to its economic impact, BBD is correlated with declines in primary productivity, biodiversity, and sustainability within managed and unmanaged forests [8,9,10].

American beech is a shade-tolerant mesophytic species, and the only species of the *Fagus* genus native to North America [11]. The ability of beech to thrive in low-light environments is partly attributed to low leaf respiration rates and the rapid responsiveness of stomata to increases in light availability [12]. Beech stomata are more responsive than co-occurring species such as red maple (*Acer rubrum* L.), red oak (*Quercus rubra* L.) and yellow poplar (*Liriodendron tulipifera* L.) [13]. From a reproductive perspective, beech seed crops are produced at 2–8-year intervals, with thousands of seeds produced each cycle (average 3,500 kg seed tree^-1^) [14]. Most of the seed falls to the ground beneath the parent tree, with local dispersal facilitated by rodents and long-range dispersal by blue jays (*Cyanocitta cristata* L.), which can carry seeds up to 4 km away from parent trees [15]. In addition to sexual reproduction, American beech readily regenerates vegetatively through root suckering, a trait increasingly observed under stress conditions, such as through disease and root injury or stem damage [16,14]. Beech trees may also regenerate vegetatively as stump sprouts following harvest or mortality. Collectively, these traits provide American beech with a competitive advantage in specific environments, particularly those characterized by disturbance or stress.

Beech bark disease progresses in three phases: the advance front, the killing front, and the aftermath forest [6]. The disease begins when scale insects colonize beech trees and feed on phloem and phelloderm cells, creating entry points for fungal infection [1,17,18]. As scale populations grow—reaching densities of up to 270 individuals cm^-2^ [19] they facilitate the establishment of pathogenic fungi (*N. faginata* and/or *N. ditissima*) [20]. The endemic scale insect, *Xylococculus betulae* (Perg.) Morrison, has also been shown to predispose trees to infection [21,22]. As parasitism by colonizing scale insects intensifies over time, trees become increasingly vulnerable to infection by necrotrophic fungi, leading to killing front conditions.

The killing front is characterized by extensive bole and stem cankering, structural decline, and mortality driven by the aggressive activity of *N. faginata* and *N. ditissima* [2]. Mortality of mature beech trees can reach up to 50% within 10 years of killing front conditions, with some stands experiencing mortality rates as high as 85% [7,1]. As susceptible trees are lost, populations of beech scale and *Neonectria* decline, and affected areas transition into the aftermath forest stage.

In the aftermath forest, vegetative regeneration often results in the formation of dense beech thickets [23,24,[25]], which can significantly impact forest structure and functions [8,9,10]. These conditions are often characterized by reduced plant biodiversity, and the suppression of recruitment and growth of more economically desirable species such as sugar maple [9,2]. Beech infection and mortality may still occur in aftermath forests, but at a lower rate than during the killing front [3]. Scale densities of fewer than 40−70 scales cm^-2^ have been reported in aftermath stands [21], with an associated decline in *Neonectria* lesions observed on surviving trees [26,24)] Reinfection may also occur in aftermath forests, along with the development of secondary killing fronts, particularly when climatic conditions allow beech scale to colonize areas where previously inhibited [27]. In North America, the aftermath zone currently encompasses most of the BBD-affected range [3,23,28], with the exception of the western and northern portions of Quebec’s beech range, where cold winter temperatures may limit scale insect survival [29].

The timeline for a forest stand to transition from the advance front to aftermath conditions remains difficult to predict, as it is influenced by a combination of host susceptibility, pathogen dynamics, and environmental factors [3]. However, research on the BBD pathosystem suggests that killing front conditions typically emerge 15–20 years after the arrival of scale insects, with mortality reaching 50% within the following decade [3,1,2]. A full transition to aftermath conditions may therefore take 20–30 years. For detailed accounts of each stage, see Ehrlich (1934), Shigo [30], Cale et al. [3], and Morrison et al. [31].

The frequency of BBD-resistant individuals (i.e., those exhibiting resistance to scale insect colonization) appears to vary regionally. For instance, Houston [2] reported that the prevalence of resistance among mature American beech was below 1% during the killing front stage. In contrast, Taylor et al. [32] documented putative resistance in 1.0-5.7% of individuals across surveyed sites in eastern Canada. Regional variation also extends to reproductive strategies among mature beech trees, which implications for forest dynamics post-BBD infestation. Specifically, previous studies have noted a partial shift from sexual reproduction via seedling establishment to an increased reliance on vegetative reproduction in disturbed areas [33], especially in regions with harsher climatic conditions [34]. Northern hardwood forests, for example, may promote root suckering, especially under baseline disturbance regimes associated with commercial forestry activities.

In Ontario, Canada—the focus of our study—beech scale was first reported in 1981 [35] and has since expanded across the entire provincial range of American beech, resulting in widespread mortality among mature trees [36]. In certain Ontario forests, timber losses due to BBD are projected to surpass $5 million by 2025 [37]. Indeed, mounting losses throughout Ontario’s managed forest zones have driven the adoption of beech-centric silvicultural systems. Notably, in 2021, several Forest Management Units (FMUs) overseeing hardwood forest regions initiated formal planning for the salvage harvesting of American beech (OMNRF, 2021). However, in post-salvage harvest areas, forestry professionals have raised concerns about the rapid establishment of dense beech thickets in the understory.

Mechanical and chemical controls are the primary supplemental interventions used alongside silvicultural systems to manage beech regeneration. Mechanical removal can be effective in the short term; however, these activities often stimulate prolific root suckering, inadvertently increasing beech stem density over time [2,38; ]. Among chemical control strategies, broadcast foliar herbicide treatments can reduce regeneration pressure at the stand scale, but pose ecological risks to mixed species hardwood systems due to potential non-target effects [39]. Stem injections of herbicides are effective for selectively controlling beech in mixed species stands (Knight et al., 2012; DiGregorio et al., 2020), however, operational challenges often limit their use. In contrast, direct spray applications to harvested stumps and standing juvenile trees—the focus of our work here—represent a more feasible option in many forest management settings. Though the efficacy and impact of this management approach on beech regeneration, and ultimately forest structure and function, are not well documented [40,41,42].

In this study, we investigate the effects of post-harvest herbicide treatments on beech regeneration over five years. Specifically, we address the following research questions: (1) To what extent does herbicide application to beech stumps (targeting the vascular cambium) and standing juvenile trees effectively suppress vegetative regeneration? (2) Does this treatment promote the recruitment of desirable species, such as sugar maple and yellow birch? (3) Does the treatment alter tree species diversity and overall forest community composition? (4) Does the timing of treatment (summer vs. fall) influence treatment efficacy in suppressing beech regeneration? (5) Does this treatment support achievement of post-harvest regeneration success criteria outlined in regional silvicultural guidelines? Collectively, our investigations aim to inform forest management strategies that mitigate ecological and economic impacts of BBD.

## Methods

**Fig 1:**
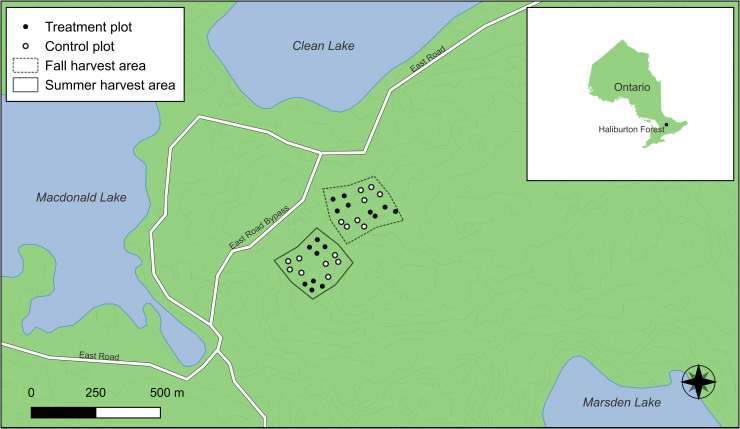
Location of Haliburton Forestin Ontario, Canada, with the plot-level spatial extent of the study area. Black dots indicate treatment plots and white dots indicate control plots. Layers were derived from Ontario GeoHub, the United States National Ice Center, and handheld GPS field data.

An experimental field study was conducted in a managed forest in Haliburton County, Ontario, Canada, to investigate the efficacy of glyphosate treatments in reducing American beech regeneration. The Haliburton Forest & Wild Life Reserve Ltd. (Fig 1) has been managed on an approximate 20-year harvest cycle since the 1980s primarily using single-tree selection; prior to that, there was a period of diameter limit cutting [43]. Beech scale was first reported at the site in 2009.

Haliburton Forest is situated within the Great Lakes-St. Lawrence Forest Region, extending across central Ontario from the St. Lawrence River to Lake Huron and west of Lake Superior (Fig 1). This region is broadly characterized by a mix of coniferous and deciduous species. Haliburton Forest lies within the Algonquin Ecodistrict, defined under Ontario’s classification system based on landscape-scale productivity patterns [44]. The regional climate is characterized as cool-temperate and humid, with an average annual temperature of 4.98 °C and average annual precipitation of 1,131 mm yr ⁻ ¹ (Environment and Climate Change Canada, 2006). The area is subject to significant NO_3-_ and NH_4+_ wet deposition, receiving some of the highest annual rates of N deposition in eastern North America (ECCC, 2023; Ro & Vet, 2023). Soils in the area are of glacial origin (Sherborne till), with upland areas consisting primarily of shallow brunisols or juvenile podzols [43]. The forest lies on Precambrian bedrock composed of undifferentiated metamorphic and igneous rock (e.g., granodiorite, tonalite, monzogranite, syenogranite, derived gneisses and migmatites) (Ontario Geological Survey, 2000; 2011).

Our study site was situated east of MacDonald Lake and south of Clean Lake, parallel to a road bypass, with an elevation of between 400−460 m above sea level. The site slopes north-northwest toward the lakes and was characterized by hardwood-dominated stands, where beech was the dominant species by basal area (15.04 ± 1.26 m^2^ ha^-1^), exceeding that of sugar maple (6.05 ± 1.38 m^2^ ha^-1^). Other species present in lower abundances include yellow birch (*Betula allegheniensis* Britt.), red maple (*A*. *rubrum*), ironwood (*Ostrya virginiana* [Mill.] K. Koch), striped maple (*A*. *pensylvanicum* L.) and eastern hemlock (*Tsuga canadensis* [L.] Carrière). Understory vegetation is dominated by sugar maple and beech, with a high abundance of raspberry (*Rubus* spp.), alongside yellow birch and striped maple, and elderberry (*Sambucus canadensis* L.), hobblebush (*Viburnum lantanoides* Michx.).

At our study site, two salvage harvests were conducted in a BBD-affected forest stand in July and October 2019. The area was designated for harvest to recover value from BBD-infected trees, based on a pre-harvest field survey (2019) indicating that all American beech trees >16 cm diameter at breast (dbh) within the stand were infested with scale insects. Pre-harvest basal area averaged 26.88 ± 0.98 m^2^ ha^-1^, with beech representing over 40% of the mature tree basal area (15.04 ± 1.26 m^2^ ha^-1^) across the site. The salvage harvests removed all American beech trees ≥20 cm dbh, reducing the stand basal area to 8.1 ± 1.1 m^2^ ha^-1^. No specific treatment was applied to remove the understory beech component prior to harvest; however, skidder operators were instructed to preferentially conduct operations along paths dominated by beech regeneration.

Each harvest area contained four 1 ha treatment blocks: two that received post-harvest glyphosate applications, and two untreated control blocks. Four circular sampling plots (radius = 10 m, area = 314 m^2^) were nested within each sample block (Fig 1). Sample locations were selected such that the outer edge of the circular plot was at least 20 m away from the edge of each harvest block, and the distance between plots (i.e., distance within and between treatment and control groups) was > 20 m to ensure spatial interspersion of samples. Salvage harvests were applied uniformly, resulting in the removal of all mature beech, while a concurrent stand improvement prescription was implemented for non-beech species.

In the treatment plots, a 50% solution of VisionMAX Silviculture Herbicide, containing 540 grams of glyphosate (N-(phosphonomethyl)glycine) acid equivalent per liter, was applied to beech stumps. The solution was diluted (1:1 with water) and applied to cambium tissue using a spray applicator, targeting the outer two inches of the stump. Standing beech trees between 16–20 cm dbh were treated using the hack-and-squirt method, in which herbicide was sprayed directly onto the cambium through cuts made with a hatchet on the tree bole. Blue dye was added to the solution to ensure beech in the treatment blocks received a sufficient volume of herbicide (~20 ml) to their cambial tissues without causing runoff, as excess can lead to unnecessary soil exposure and potential non-target effects [40]. The herbicide solution was applied by a licensed exterminator using a spray applicator within 48 hours of each harvest. The treatment timing aligns with literature suggesting that glyphosate application within 96 hours can translocate through root grafts, effectively killing adjacent stems (i.e., root suckers) connected to the parent tree [41]. The environmental conditions during the applications were dry, helping to control the potential influence of precipitation-related effects on chemical sorption.

For each sampling period, we measured the abundance of tree species at each plot across five size class categories: (1) <2 cm dbh (and >1.3 m in height); (2) 2.0–7.9 cm dbh; (3) 8.0–15.9 cm dbh; (4) 16.0–19.9 cm dbh; and (5) ≥ 20 cm dbh. Mature trees (i.e., size class 5) had their dbh recorded and were classified as either acceptable growing stock (AGS) or unacceptable growing stock (UGS), as per the Ontario Tree Marking Guidelines [45]. To ensure accuracy during sampling, plot boundaries were marked with flagging tape, and each plot was divided into four quadrats (NE, NW, SE, SW) using tape measures aligned with cardinal directions. Pre-harvest data were collected from June to July 2019 and post-harvest data were collected at 1-year (June 2020) and 5-years (August 2024) post-harvest.

All surveyed trees were classified into one of three categories based on forest management objectives: (1) target species, (2) acceptable species, and (3) nonacceptable species. These categories were informed by the forest management objectives outlined in the region’s silvicultural guidelines [46]. Target species are prioritized for conservation and management based on their ecological or economic value. In our study, these included: sugar maple, yellow birch, red oak, black cherry (*Prunus serotina* Ehrh.), and eastern white pine (*Pinus strobus* L.). Acceptable species were considered to have beneficial or neutral ecological or economic value and included: white birch (*Betula papyrifera* Marsh.), balsam fir (*Abies balsamea* [L.] Mill.), bigtooth aspen (*Populus grandidentata* Michx.), eastern hemlock, black spruce (*Picea mariana* [Mill.] B.S.P.), white spruce (*Picea glauca* [Moench] Voss), red maple, and trembling aspen (*Populus tremuloides* Michx.). Nonacceptable species are considered less desirable due to their competitive growth patterns or limited ecological benefits relative to forest management objectives. These species included: American beech, striped maple, pin cherry, hobblebush, elderberry, dogwood (*Cornus* spp.), and ironwood.

Prior to the salvage harvests, the plots exhibited similar stand characteristics, as indicated by the pre-harvest site summary data (Table 1). Specifically, we applied the Shannon and Simpson indices— two measures of community diversity that account for both the number of species (richness) and their relative abundance (evenness) [47]. A Kruskal-Wallis test of plot-level Shannon and Simpson indices revealed no statistically significant differences in community diversity between treatment and control plots prior to harvest in 2019 (*p* = 0.68 and *p* = 0.70, respectively).

**Table 1:** Pre-harvest (2019) summary values for 32 research plots used to evaluate Beech bark disease regeneration treatments. Data here represents mean values for 32 research plots, evenly distributed as 16 treatment and 16 control plots. American beech is categorized into five size classes: size class 1 (<2 cm dbh), size class 2 (2.0–7.9 cm dbh), size class 3 (8.0–15.9 cm dbh), size class 4 (16.0–19.9 cm dbh), and size class 5 (≥20 cm dbh). Tree counts were also categorized according to a forest management classification system, with each tree assigned to one of three groups: (1) target species, (2) acceptable species, or (3) nonacceptable species.

Size class	Treatment vs. Control	Absolute beech count *(n)*	Relative abundance of beech	Species richness	Target species count *(n)*	Acceptable species count *(n)*	Nonacceptable species count *(n)*
1	C	523	0.81	8	93	6	24
1	T	514	0.71	8	156	14	35
2	C	324	0.46	11	240	31	109
2	T	186	0.3	10	250	15	159
3	C	23	0.14	14	78	22	39
3	T	15	0.13	8	59	8	35
4	C	6	0.29	7	5	4	6
4	T	6	0.2	8	15	4	5
5	C	68	0.49	10	47	20	3
5	T	79	0.7	8	25	6	3

### Statistical analyses

All statistical analyses were conducted using R v. 4.2.2 (R Foundation for Statistical Computing, Vienna, Austria). First, for all plots we used the ‘vegan’ R package [48] to compute Shannon and Simpson diversity indices. Then, to assess the effects of both cut stump treatment and time on absolute beech counts and tree diversity metrics, we applied a linear mixed-effects model with treatment (cut stump herbicide application vs. untreated control), time, and their interaction as fixed effects, while accounting for the nested structure of the study design by including plot identity as a random effect within block identity. The hierarchical structure accounted for potential spatial non-independence by modeling similarities among plots within the same block. To evaluate the effect of seasonal treatment timing, harvest season (summer vs. fall) was included as a fixed effect, along with a harvest season-by-treatment interaction term. Additionally, the dataset was partitioned by harvest season to allow for independent analyses.

Model fitting was performed using the ‘lme4’ package [49], with model performance evaluated using the ‘MuMIn’ package [50] to compute the marginal and conditional *r*^2^ values, which quantify the variance explained by fixed effects alone and by both fixed and random effects, respectively. Then, pairwise comparisons of estimated marginal means (EMMs) among treatment groups over time were conducted using the ‘emmeans’ package [51]. In all of our mixed effects model analyses and diagnostics, for variables exhibiting zero-inflation (i.e., count data with an excess of zero values), we applied the ‘NBZIMM’ package [52] in order to accommodate zero-inflated negative binomial distributions; the decision to account for zero inflation was based on visual diagnostics and goodness-of-fit statistics (Table 2). Finally, we examined spatial autocorrelation among residuals of the dependent variables using Moran’s eigenvector maps (MEMs), implemented in the ‘ape’ package [53], to quantify potential spatial structure affecting model outcomes. To support practical interpretation of the results in a manner consistent with forest management guidelines, we scaled all reported data and figures to a per-hectare basis.

**Table 2:** Tree abundance and diversity metrics across 32 research plots used to evaluate Beech bark disease regeneration treatments in 2019 (pre-harvest), 2020 (1-year post-harvest), and 2024 (5-years post-harvest). American beech was grouped into five size classes, and tree counts were also categorized by management relevance (target, acceptable, nonacceptable). Values in parentheses represent the 95% confidence interval around each estimated marginal means (based on mixed-effects models).

		Pre-harvest (stems ha^-1^)		1-year post-harvest (stems ha^-1^)		5-year post-harvest (stems ha^-1^)		Model Diagnostics	
Variable	Zero Inflated	C	T	C	T	C	T	Marg. *r*^*2*^	Cond. *r*^*2*^
Total abundance (*n*) beech	No	1878.0 (1212.7, 2543.2)	1591.5 (897.6, 2285.4)	1069.5 (407.4, 1734.7)	703.4 (9.5, 1397.3)	2387.3 (1722.1, 3052.6)	1209.6 (515.7, 1903.5)	0.36	0.67
Abundance *(n)* of beech stems <2 cm dbh	No	1040.8 (585.7, 1492.8)	1021.7 (563.4, 1496.0)	814.8 (359.7, 1266.8)	592.0 (117.8, 1066.3)	1741.2 (1286.0, 2193.2)	904.0 (432.9, 1378.3)	0.26	0.63
Abundance *(n)* of beech stems 2.0–7.9 cm dbh	Yes	646.1 (391.5, 897.6)	369.2 (105.0, 636.6)	229.2 (−28.6, 483.8)	98.7 (−171.9, 366.0)	576.1 (321.5, 830.8)	286.5 (19.1, 555.9)	0.34	0.60
Abundance *(n)* of beech stems 8.0–15.9 cm dbh	Yes	60.5 (12.7, 111.4)	50.9 (−3.1, 108.2)	9.5 (−44.6, 63.7)	31.83 (−28.6, 89.1)	111.4 (54.1, 171.9)	31.8 (−22.3, 85.9)	0.28	0.67
Abundance *(n)* of beech stems 16.0–19.9 cm dbh	Yes	15.9 (0.0, 35.0)	12.7 (−3.2, 28.6)	15.9 (0.0, 35.0)	0.0 (−15.9, 15.9)	15.9 (0.0, 31.8)	0.0 (−15.9, 15.9)	0.11	0.72
Abundance *(n)* of beech stems ≥20 cm dbh	Yes	136.9 (101.9, 168.7)	156.0 (121.0, 194.2)	(19.1 (−15.9, 57.3)	9.5 (−31.83, 50.9)	19.1 (−15.9, 57.3)	9.5 (−31.8, 50.9)	0.64	0.97
Target species count (*n*)	No	28.9 (−1.7, 59.5)	31.6 (−0.4, 63.5)	26.8 (−3.8, 57.4)	37.4 (5.5, 69.4)	1753.9 (779.9, 2727.9)	2333.2 (1317.8, 3351.8)	0.25	0.68
Acceptable species count (*n*)	Yes	5.4 (−4.0, 14.7)	4.4 (−5.5, 14.3)	6.8 (−2.8, 16.4)	7.3 (−2.6, 17.3)	483.8 (184.6, 783.0)	429.7 (127.3, 735.3)	0.10	0.63
Nonacceptable species count (*n*)	No	70.3 (49.5, 91.1)	64.8 (43.9, 85.6)	59.5 (38.7, 80.3)	74.3 (53.5, 95.1)	3444.1 (2782.0, 4109.4)	3211.7 (2546.5, 3873.8)	0.30	0.43
Species richness	No	6.8 (5.3, 8.4)	6.1 (4.5, 7.7)	9.3 (7.7, 10.8)	8.5 (6.9, 10.1)			0.33	0.66
Shannon diversity index	No	1.1 (0.9, 1.3)	1.2 (1.0, 1.4)	1.6 (1.4, 1.8)	1.7 (1.5, 1.9)			0.48	0.77
Simpson diversity index	No	0.6 (0.5, 0.64)	0.6 (0.5, 0.7)	0.7 (0.6, 0.8)	0.8 (0.7, 0.82)			0.45	0.71

## Results

**Fig 2:**
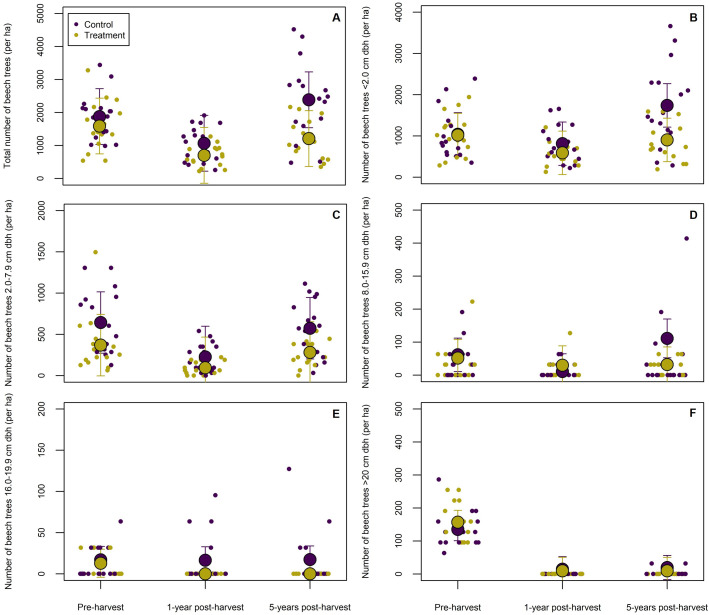
Estimated marginal means of plot-level beech abundance from pre-harvest to 5-year post-harvest: (A) total beech count across all size classes, (B) number of stems in size class 1 (<2 cm dbh), (C) number of stems in size class 2 (2.0–7.9 cm dbh), (D) number of trees in size class 3 (8.0–15.9 cm dbh), (E) number of trees in size class 4 (16.0–19.9 cm dbh), and (F) number of trees in size class 5 (≥20 cm dbh). Large circles with error bars represent the estimated marginal means (± 95% CI), while smaller points indicate individual plot-level observations. Values were derived from a linear mixed-effects model, with cut stump herbicide treatment (vs. untreated control) specified as a fixed effect and random effects accounting for variation at the block and plot levels. Corresponding values for each estimated marginal mean are provided in Table 2.

**Fig 3:**
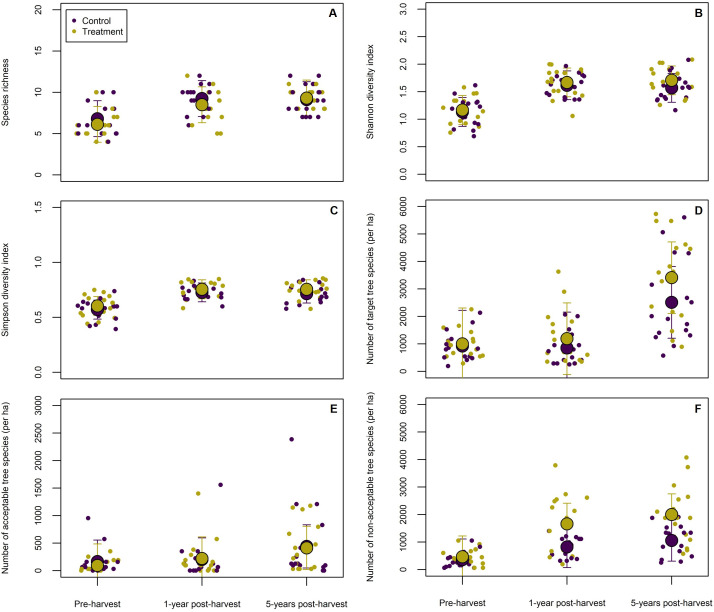
Estimated marginal means of diversity and forest management metrics from pre-harvest to 5-years post-harvest: (A) species richness, (B) Shannon diversity, (C) Simpson diversity, (D) abundance of target species, (E) abundance of acceptable species, and (F) abundance of nonacceptable species. Large circles with error bars represent estimated marginal means (± 95% CI), while smaller points indicate individual plot-level observations. Values were derived from a linear mixed-effects model, with cut stump herbicide treatment (vs. control) specified as a fixed effect and random effects accounting for variation at the block and plot levels. Corresponding values for each estimated marginal mean are provided in Table 2.

The application of glyphosate to cut stumps and juvenile beech trees led to a significant reduction in sapling abundance (size class 1; < 2 cm dbh) by 5-years post-harvest (*p* < 0.001; Table 2, Fig 2), indicating effective suppression of beech regeneration. At this time, plots treated with glyphosate contained an average of 904 stems ha ⁻ ¹ (95% CI: 433−1,378 stems ha^-^¹), compared 1741 stems ha^-^¹ (95% CI: 1,286−2,193 stems ha ⁻ ¹) in untreated control plots (Table 2, Fig 2-B). No significant differences were detected between treatment and control plots at 1-year post-harvest (*p* = 0.345). The marginal *r*^*2*^ = 0.26 and conditional *r*^*2*^ = 0.63 indicate that random effects—specifically, block- and plot-level spatial structure—accounted for a considerable, but not all, of the variation in beech sapling abundance over time (Fig 3).

For larger beech saplings (size class 2; 2.0-7.9 cm dbh), the results approached significance, with 287 stems ha^-1^ (95% CI = 19−556 stems ha^-1^) observed in treatment areas vs. 576 stems ha^-1^ (95% CI = 322−831 stems ha^-1^) in controls areas after five years (*p* = 0.07). Here, the interaction effect between treatment and time at 5-years post-harvest was not significant (*p* = 0.89). A significant temporal decline in larger sapling abundance was observed at 1-year post-harvest (*p* < 0.001), but no effect was observed 5-years post-harvest (*p* = 0.36). The divergence between the marginal *r²* = 0.34 and conditional *r²* = 0.60 indicates the influence of random effects due to block- and plot-level spatial variation in the beech sapling distribution.

Cumulative beech counts (across all size classes) were lower in treatment plots at both 1- and- 5-years post-harvest (*p* < 0.001 and *p* < 0.01, respectively). By 5-years post-harvest, 1,210 stems ha^-1^ (95% CI = 516−1,904 stems ha^-1^) were predicted in treatment plots, compared to 2,387 stems ha^-1^ (95% CI = 1,722−3,053 stems ha^-1^) in control plots. A significant interaction effect between treatment and time was detected 5-years post-harvest (*p* < 0.001), but not 1-year post-harvest (*p = *0.75). Cumulative beech counts explained more variability (marginal *r²* = 0.36; conditional *r²* = 0.67) than individual size classes, with the exception of beech trees >20 cm dbh.

The season in which glyphosate treatments were applied (i.e., summer vs. fall) had no significant effect on beech sapling abundance by 5-years post-harvest (*p *= 0.398). Partitioning the dataset by treatment season showed minor differences in treatment efficacy between seasons. Specifically, summer cut stump treatments significantly reduced beech regeneration (size class 1) by 5-years post-harvest (*p* < 0.01), with a significant treatment-by-time interaction effect (*p* < 0.01). At this time, summer-treated harvest plots contained 573 stems ha ⁻ ¹ (95% CI: −850−2,738 stems ha ⁻ ¹) compared to 1,763 stems ha ⁻ ¹ (95% CI: 803−2,722 stems ha ⁻ ¹) in control areas. Fall treatments were also effective in suppressing beech regeneration by the 5-year mark (*p* < 0.01), with treatment areas averaging 1,238 stems ha ⁻ ¹ (95% CI: −264−2,738 stems ha ⁻ ¹) compared to 1,719 stems ha ⁻ ¹ (95% CI: 707−2,289 stems ha ⁻ ¹) in control areas. However, the treatment × time interaction for fall treatments only approached statistical significance (*p* = 0.09).

By 5-years post-harvest, a significant increase in the abundance of target species was observed in treatment plots, averaging 2,333 stems ha ⁻ ¹ (95% CI: 1,318−3,352), compared to 1,754 stems ha ⁻ ¹ (95% CI: 780−2,728) in control plots. The increase in target species within treatment plots was primarily driven by sugar maple (relative abundance [RA]=77.4%) and yellow birch (RA = 22.3%). In control plots, sugar maple (RA = 74.8%) and yellow birch (RA = 24.4%) similarly accounted for most of the observed target species abundance. The marginal *r²* = 0.25 and conditional *r²* = 0.68 indicate that random effects (i.e., block- and plot-level spatial structure) explained a significant portion of the observed variation (Table 2).

By 5-years post-harvest, the abundance of non-acceptable species increased in both treatment and control plots: 3,212 stems ha^-1^ (95% CI: 2,547−3,874 stems ha^-1^) and 3,444 stems ha^-1^ (95% CI: 2,782−4,109 stems ha^-1^), respectively. No significant treatment effect was detected at this time (*p* = 0.887), indicating similar trajectories between treatment and control plots. Increases in nonacceptable species across the study area were primarily driven by beech saplings, with relative abundances that were nearly twice as large in control (RA = 56.7%) vs. treatment plots (RA = 28.2%). In treatment plots, pin cherry (RA = 27.4%) was the co-dominant nonacceptable species, followed by lower abundances of striped maple (RA = 17.0%) and elderberry (RA = 16.3%) by 5-years post-harvest. Similarly, pin cherry (RA = 19.7%) and striped maple (RA = 8.3%) were also observed in high proportions across control plots. No significant differences in tree diversity—measured by Shannon and Simpson indices and species richness—were observed between treatment and control groups across all time periods. The application of Moran’s eigenvector maps (MEMs) confirmed the absence of spatial autocorrelation among the sample plots (S1 Table).

## Discussion

Our results indicate that after five years, glyphosate treatments in harvest areas effectively suppressed vegetative regeneration of American beech, while facilitating the successional advancement of co-occurring hardwood species. At this time, the emergence of small beech saplings (<2.0 cm dbh) was reduced by 48% in treatment plots, with an estimated density of 904 stems ha^-1^ compared to 1,741 stems ha^-1^ in untreated control plots. When combining all size classes, total beech abundance declined significantly in treatment plots (1,210 stems ha^-1^) relative to controls (2,387 stems ha^-1^). The abundance of target species, such as sugar maple and yellow birch, was significantly higher in treatment plots, while no observable treatment effects were detected for acceptable or nonacceptable species.

Our findings are consistent with previous studies demonstrating the efficacy of cut stump and hack and squirt methods in controlling beech regeneration [40,41,42]. Specifically, the significant divergence in sapling abundance between treatment and control plots by 5-years post-harvest indicates a sustained effect of the treatment on beech regeneration dynamics. In contrast, salvage harvest prescriptions that exclude herbicide treatments may inadvertently promote increased understory and mid-story beech abundance through both the emergence of new vegetative sprouts and the accelerated growth of preexisting root suckers [ 54,55]. Indeed, plot-level assemblages of nonacceptable species at untreated control sites were dominated by beech after five years. This direct application method also minimizes the potential for non-target effects associated with alternative approaches, such as broadcast foliar spraying, which has also been shown to suppress beech regeneration effectively albeit with non-trivial impacts on non-target species [56].

While no significant effect of treatment season (summer vs. fall) on beech regeneration was detected after five years, summer-treated plots averaged 668 stems ha ⁻ ¹ fewer than fall-treated plots. When partitioned by season, only the summer applications exhibited a significant treatment-by-time interaction, suggesting that glyphosate applied in July produced a stronger suppression effect. The observed seasonal difference may be attributable to the phenological and physiological characteristics of target trees. For instance, trees cut in the fall generally possess greater root energy reserves, facilitating enhanced resprouting the following season. Alternatively, if summer-treated trees allocate fewer resources to their root systems and more to active growth, aboveground tissues are likely to be more susceptible to mechanical damage from skidder operations. While these underlying physiological mechanisms remain an area of future scientific enquiry, our results demonstrate that cut-stump and hack-and-squirt treatments are effective throughout the peak operational season for commercial forestry [57].

Comparing observed regeneration trends to regional silvicultural guidelines offers an additional basis for evaluating treatment effectiveness. Our results exceeded the regional silvicultural guideline of 805 stems ha^-1^ for hardwood shelterwood harvests [58], with 2,333 stems ha^-1^ of target species observed in glyphosate-treated plots by 5-years post-harvest, compared to 1,754 stems ha^-1^ in control plots. The abundance of target species observed after five years also surpassed the standard threshold of 1,325 stems ha^-1^ for shelterwood and selection harvest systems in similar forest types [58,59] (though no formal benchmarks currently exist for salvage harvest scenarios targeting beech). It is important to note that our stem counts included all individuals ≥1.3 m in height, whereas silvicultural guidelines typically apply a ≥ 2 m threshold for hardwood species. As a result, the interpretation that regeneration standards were well-exceeded based on our control method, should be made cautiously. Furthermore, our results may partially reflect the regenerative contributions of ground-layer vegetation—seedlings and sprouts established beneath the pre-harvest understory community. This regenerative cohort may respond rapidly following overstory removal, and quantifying these pre-existing conditions in future studies—such as through finely stratified sampling of understory layers by species and height—could deepen our understanding of treatment effects on successional dynamics. For example, similar patterns of dense beech sapling recruitment have been observed in selection-harvested northern hardwood stands in the Great Lakes region, where small sapling layers were often dominated by beech and suppressed the establishment of other desirable species [25]. Such findings underscore the importance of accounting for pre-existing regeneration structure when evaluating treatment outcomes.

In our study, the salvage harvest of mature beech trees altered overstory species composition and contributed to stand-level structural changes; however, at five years post-harvest, understory community assemblages remained largely unchanged. These findings align with those of Dracup and MacLean [60], who reported that the targeted removal of mature beech did not significantly influence species composition or regeneration after 10 years, and emphasized that rapid regrowth from beech sprouts conferred a strong competitive advantage. This pattern is further supported by evidence indicating that, under commonly applied silvicultural systems, and in the absence of supplemental controls, initial stand conditions exert greater influence on regeneration composition than specific treatments [61]. Similarly, Searle et al. [62] found that while tending treatments can temporarily reduce beech regeneration densities, sugar maple regeneration remains below stocking targets even under uniform shelterwood systems, indicating that beech retains a competitive advantage in post-disturbance environments. We suggest that shifts in overall tree assemblages were not detected in our study because measures of evenness and richness were spatiotemporally constrained. Specifically, the study area is characterized by homogeneous physical features (e.g., moisture, slope), vegetation, and substrate types, and the results presented here are based on preliminary 5-year follow-up data. Shelterwood systems are also recognized for maintaining species diversity, which may contribute to the observed structural uniformity.

The costs associated with glyphosate treatment methods have been estimated to range from $39.43 to $62.34 USD acre^-1^ [40]. However, actual implementation costs are often higher in practice. In many cases, herbicide application is contracted to external service providers, with expenses typically ranging from $500−1,000 CAD ha^-1^. Alternatively, forestry operations may employ a licensed exterminator on staff, in which case costs are primarily associated with salary, equipment, and herbicide procurement. While this approach avoids potentially broader ecological disturbances associated with other methods (e.g., broadcast foliar spraying), these additional logistical and operational factors must be carefully considered when evaluating the economic feasibility of integrating this forest management strategy.

Despite the availability of effective management practices, predicting the longer-term response of northern hardwood forests affected by BBD remains challenging due to complex interactions between the disease, host tree resilience and functional traits, and environmental variables [reviewed by 3]. The distinctive climatic and topographical features of our study region [63] are expected to exert regional influences on BBD severity and spread. For example, variation in elevation, soil properties, and moisture retention directly influences root function and host stress, with prior studiesindicating that site- and moisture-related stress affects defense allocation and tree susceptibility to biotic agents [64,65]. Environmental conditions such as nutrient-poor soils, particularly those low in N and P, have been linked to greater susceptibility to *N. ditissima* infection [66], while climate patterns (e.g., dry conditions promoting canker expansion and autumn rainfall reducing disease severity) further complicate long-term BBD dynamics [23,67]. Additionally, microsite features like slope and aspect can affect infection severity, with western-facing trees on steeper slopes exhibiting reduced *Neonectria* infections compared to eastern-facing trees on flatter ground [68,69]. Collectively, existing data suggest that idiosyncratic climatic and topographical characteristics play a critical role in shaping long-term forest resilience to BBD. However, our study indicates that glyphosate treatments exert short-term, and potentially longer-term, effects in governing ecological dynamics in BBD-affected forests.

## Supporting information

S1 Table Results of the Moran’s Eigenvector spatial analysis of tree abundance and diversity metric analyses from pre-harvest to 5-year post-harvest. (DOCX)

S2 Table Summary data supporting the analyses presented in the main text. (DOCX)
