# Supplementary material for: Glyphosate treatments for managing successional dynamics in beech bark disease-affected northern hardwood forests
Source: PLoS One. 2025 Nov 14;20(11):e0336126. doi: 10.1371/journal.pone.0336126 (PMC12617849; doi:10.1371/journal.pone.0336126)
Supplement: S1 Table — (DOCX) [file pone.0336126.s001.docx]

**Supplementary Materials**

**S2 Table.** Summary data supporting the analyses presented in the main text.
