# Supplementary material for: Glyphosate treatments for managing successional dynamics in beech bark disease-affected northern hardwood forests
Source: PLoS One. 2025 Nov 14;20(11):e0336126. doi: 10.1371/journal.pone.0336126 (PMC12617849; doi:10.1371/journal.pone.0336126)
Supplement: S2 Table — (DOCX) [file pone.0336126.s002.docx]

**Supplementary Materials**

**S1 Table.** Results of the Moran’s Eigenvector spatial analysis of tree abundance and diversity metric analyses from pre-harvest to 5-year post-harvest. The analysis examines the degree of spatial autocorrelation for each variable, including American beech across five size classes: size class 1 (<2 cm dbh), size class 2 (2.0–7.9 cm dbh), size class 3 (8.0–15.9 cm dbh), size class 4 (16.0–19.9 cm dbh), and size class 5 (≥20 cm dbh). We similarly assessed diversity metrics, including species richness, and Shannon and Simpson diversity indices. Tree counts were also categorized according to a forest management classification system, where each tree is assigned to one of three groups: (1) target species, (2) acceptable species, or (3) nonacceptable species. Moran’s I values range from -1 to +1, where values near -1 indicate strong negative spatial autocorrelation (dispersion), values near +1 indicate strong positive spatial autocorrelation (clustering), and values close to zero suggest spatial homogeneity (no significant autocorrelation). Values in brackets represent the associated *p*-values from the Moran’s I test, where *p*<0.05 indicates significant spatial structure.

| **Variable** | **Pre-harvest** | **1-year Post-harvest** | **5-year Post-harvest** |
| --- | --- | --- | --- |
| Total beech count | -0.040 (0.737) | -0.085 (0.019) | -0.018 (0.527) |
| Beech size class 1 | -0.033 (0.990) | -0.128 (<0.001) | -0.023 (0.675) |
| Beech size class 2 | -0.033 (0.964) | -0.044 (0.613) | -0.028 (0.864) |
| Beech size class 3 | -0.095 (0.002) | -0.025 (0.702) | -0.077 (0.005) |
| Beech size class 4 | -0.044 (0.603) | -0.051 (0.335) | -0.070 (0.020) |
| Beech size class 5 | -0.009 (0.297) | N/A | -0.045 (0.452) |
| Species richness | -0.027 (0.806) | -0.038 (0.784) | -0.043 (0.641) |
| Shannon diversity | -0.017 (0.503) | -0.062 (0.179) | -0.030 (0.907) |
| Simpson diversity | -0.017 (0.496) | -0.046 (0.551) | -0.030 (0.947) |
| Target species | -0.140 (0.000) | -0.134 (<0.001) | -0.050 (0.431) |
| Acceptable species | -0.032 (0.974) | -0.026 (0.748) | -0.069 (0.078) |
| Nonacceptable species | -0.029 (0.890) | -0.063 (0.165) | -0.01 (0.322) |

**S2 Table.** Summary data supporting the analyses presented in the main text.
